# Delayed Diagnosis of Sheehan’s Syndrome in an 89-Year-Old Female: A Case Report and Review of Literature

**DOI:** 10.7759/cureus.85332

**Published:** 2025-06-04

**Authors:** Basim S Samman, Abdullah F Alzarroug, Raghad Altayyar, Bayan S Alalawi, Hatim Mahmoud

**Affiliations:** 1 Internal Medicine Department, Prince Mohammed Bin Abdulaziz Hospital, Ministry of National Guard - Health Affairs, Medina, SAU; 2 Endocrinology Department, Prince Mohammed Bin Abdulaziz Hospital, Ministry of National Guard - Health Affairs, Medina, SAU

**Keywords:** adrenal insufficiency, delayed diagnosis, hyponatremia, pan-hypopituitarism, sheehan’s syndrome

## Abstract

Sheehan's syndrome, a rare consequence of severe postpartum hemorrhage, is marked by partial or complete deficiencies in pituitary hormones due to pituitary infarction. Patients may not exhibit symptoms for decades, often delaying the pursuit of medical attention. We present the case of an 89-year-old woman who exhibited symptoms of hyponatremia and adrenal insufficiency, with a history of severe postpartum hemorrhage that led to a hysterectomy 46 years ago. Her hormonal panel revealed deficiencies across all anterior pituitary gland axes. MRI findings showed a partially empty sella. To our knowledge, this case has the longest documented delay in presentation, exceeding four decades. It underscores the need to maintain a high clinical suspicion for alternative causes of hypoosmolar hyponatremia, particularly in cases unresponsive to rehydration therapy.

## Introduction

Sheehan’s syndrome was first described in 1937 by Harold L. Sheehan as an anterior pituitary gland necrosis due to severe postpartum hemorrhagic shock leading to hormonal deficiencies with various clinical presentations and significance [1]. The prevalence and incidence of Sheehan’s syndrome have been declining, particularly in countries with advanced obstetric care systems, with estimated incidence reaching up to five cases per 100,000 live births [2]. Patients may present shortly after a hemorrhagic event with a characteristic history of failure of lactation, amenorrhea, infertility, and signs and symptoms of deficient hormones [3].

The duration between the culprit event and first medical encounter is extremely variable in the literature, with rare cases presenting as late as three to four decades, depending on the degree of hormonal deficiencies [4]. For instance, a retrospective analysis of 124 patients reported an average diagnostic delay of 20.37 years, ranging from two to 36 years, with a mean age at diagnosis of 50.4 years [5]. This delay of diagnosis presents significant diagnostic challenges, particularly when nonspecific symptoms of hypopituitarism emerge decades after childbirth. Timely diagnosis and hormonal replacement are crucial to prevent severe and life-threatening complications related to the hormonal deficiencies.

## Case presentation

An 89-year-old woman from Saudi Arabia presented to the emergency department of our hospital with a chronic history of generalized fatigue and diffuse abdominal pain that markedly disabled her daily functioning, associated with significant weight loss and poor appetite. Her previous medical history was noted for uncontrolled diabetes mellitus, coronary artery disease, and bronchial asthma. On examination, she was vitally stable with a blood pressure of 137/62 mmHg, heart rate of 67 beats per minute, respiratory rate of 20 breaths per minute, temperature of 37.7° Celsius, and an oxygen saturation of 98% on room air. She was conscious, alert, and oriented, with a distended abdomen (secondary to obesity) and an old-appearing low transverse scar. Examination of other systems was unremarkable.

Initial laboratory results revealed a serum sodium of 122 mmol/L with a random blood glucose of 12.8 mmol/L; other serum electrolytes were within normal limits. She has a normocytic anemia (hemoglobin 112 g/L, mean corpuscular volume 85.4) with normal white blood cell and platelet counts. Renal and liver functions were normal.

The patient was admitted under the care of internal medicine as a case of hyponatremia. Her hyponatremia investigations showed a low serum osmolality (257 mOsm/kg), high urine osmolality (310 mOsm/kg), high urinary sodium (66 mmol/L), normal thyroid stimulating hormone (TSH) (1.39 μIU/mL), low free T4 (<5.41 pmol/L), and low random cortisol (123 nmol/L). These investigations were suspicious for a central cause for the hyponatremia. Upon further questioning, she gave a history that, when she was 43 years old, she had a severe postpartum hemorrhage requiring blood transfusion, which led to a hysterectomy following the birth of her youngest son, 46 years ago. After delivery, she was unable to lactate her baby. 

**Figure 1:**
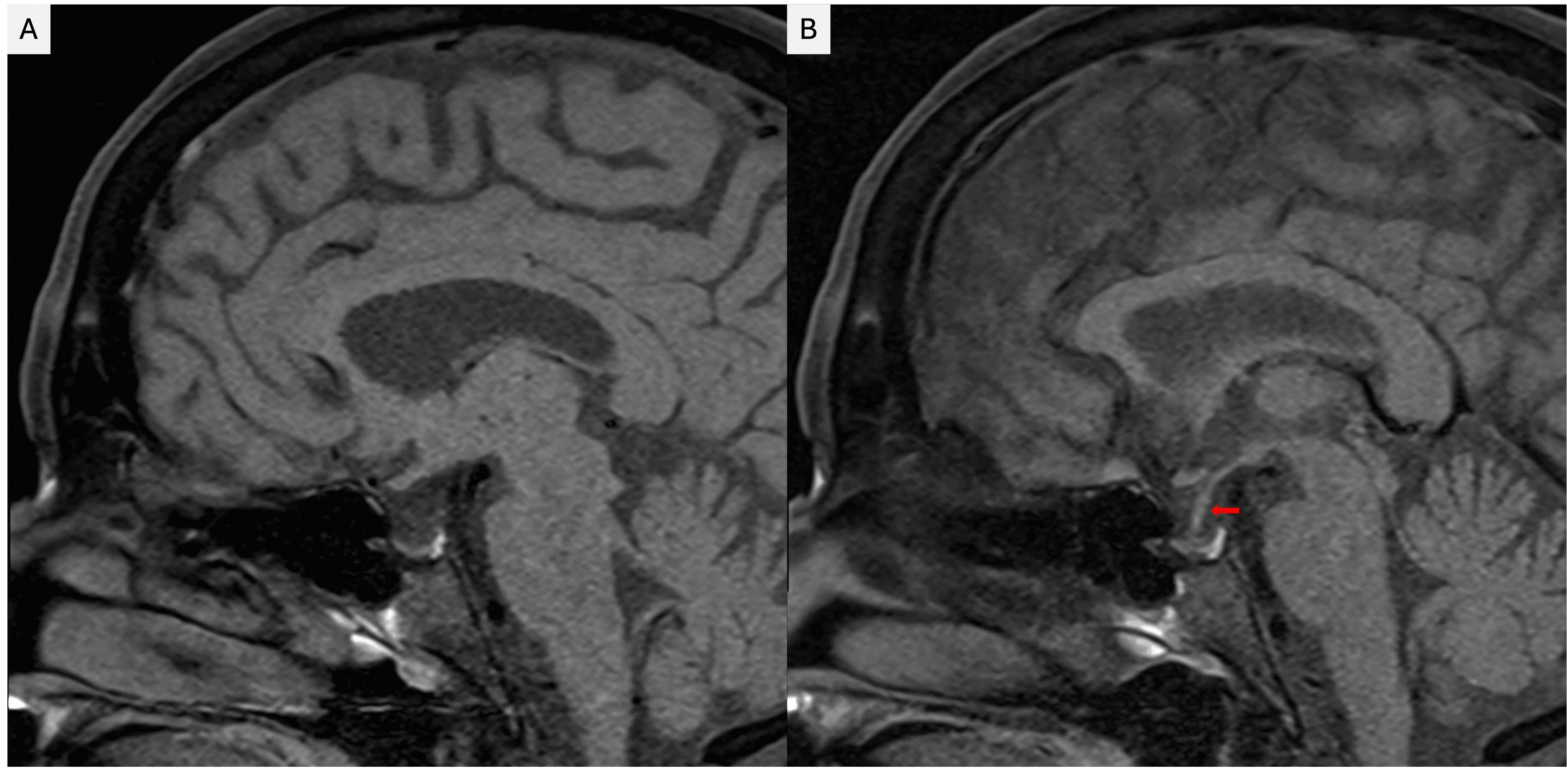
MRI of the mid-sagittal section of the brain showing (A) partially empty sella turcica; (B) Pituitary stalk (infundibulum) extends downward to the sellar floor (Red arrow).

A plasma hormonal panel revealed a picture of panhypopituitarism with deficiencies in all the axes of the anterior pituitary gland (Table 1). The patient was started on hormonal replacement therapy with hydrocortisone 10 mg in the morning and 5 mg in the evening, and levothyroxine with a weekly uptitration of 25 mcg, aiming for a maintenance dose of 75 mcg once daily. Pituitary MRI with contrast showed an enlarged sella turcica and predominantly filled with cerebrospinal fluid (CSF). The pituitary gland was significantly compressed against the sellar floor, consistent with a partially empty sella (Figure 1).

**Table 1:** Patient’s plasma hormonal panel

Plasma Hormone	Patient Value	Reference Range
Cortisol Baseline 8:00 am	55 nmol/L	102.1-535.2 nmol/L
Cortisol 30 minutes after ACTH stimulation	218.5 nmol/L	
Cortisol 60 minutes after ACTH stimulation	288.1 nmol/L.	
Adrenocorticotropic hormone (ACTH)	27.97 pg/mL	4.7-48.8 pg/mL
Thyroid-stimulating hormone (TSH)	1.39 mlU/L	0.35-4.94 mlU/L
Free T4	<5.41 pmol/L	9-19 pmol/L
Free T3	2.48 pmol/L	2.6-5.7 pmol/L
Follicle-stimulating hormone (FSH)	4.18 IU/L	26.72-144.41 IU/L
Luteinizing hormone (LH)	1.02 IU/L	10.39-64.57 IU/L
Prolactin	94.6 mlU/L	109-557 mlU/L
Insulin-like growth factor	0.7 ug/ml	2.2-4.5 ug/ml

Following treatment initiation, her symptoms improved dramatically, and laboratory tests improved significantly, with serum sodium levels reaching lower limits of normal (133-134 mmol/L) upon discharge.

## Discussion

An 89-year-old woman presented with hyponatremia, fatigue, and abdominal pain, with a history of severe postpartum hemorrhage 46 years ago. Investigations revealed panhypopituitarism due to Sheehan syndrome, a rare condition caused by ischemic pituitary necrosis following significant blood loss during or after childbirth [1].

While Sheehan syndrome is well-documented in the medical literature, this case stands out due to an extraordinary diagnostic delay of 46 years, far surpassing the maximum delay in most cases [5,6], and to the best of our knowledge, has the longest documented delay in presentation, with the previously reported maximum delay being 45 years [4]. This exceptionally prolonged delay in diagnosis likely occurred because the postpartum hemorrhage took place near the patient’s menopausal age, which inadvertently masked the hormonal deficiencies that typically manifest in Sheehan syndrome. Symptoms such as amenorrhea and failure to lactate [3], both of which often raise early suspicion, were not flagged as abnormal due to the menopausal timing of the event. Additionally, non-specific symptoms such as fatigue, abdominal discomfort, and electrolyte imbalances such as hyponatremia might have been attributed to aging or other common conditions in elderly patients, further delaying accurate diagnosis. This case highlights the importance of maintaining clinical suspicion for Sheehan syndrome as a potential diagnosis, even several decades after a history of postpartum hemorrhage. Failure to diagnose Sheehan syndrome in a timely manner may result in significant morbidity, as delayed treatment can lead to complications such as severe hyponatremia, which carries potentially life-threatening consequences [7].

The diagnosis of Sheehan's syndrome requires a high index of suspicion due to its rarity and variability of clinical manifestations [4]. The initial diagnosis is primarily based on a comprehensive patient history and physical examination, emphasizing hormone deficiency symptoms and a suggestive obstetric background [8]. This is followed by confirmation through laboratory investigations, and imaging modalities, including the measurement of anterior pituitary hormone levels and magnetic resonance imaging (MRI) of the pituitary gland [4,8]. Obstetric history of postpartum hemorrhage is a crucial factor in diagnosis; approximately 32% of patients with postpartum hemorrhage develop Sheehan's syndrome [9]. 

The clinical presentation of Sheehan’s syndrome primarily depends on the specific deficiencies in pituitary hormones, leading to a highly variable presentation [9]. Failure to lactate due to prolactin deficiency is usually the first and earliest feature of Sheehan’s syndrome [10]. In a retrospective cohort study conducted by Ramiandrasoa et al. in France, including 39 women with Sheehan's syndrome studied from 1980 to 2011, the study found that the mean diagnostic delay of Sheehan's syndrome was 2.52 ± 3 months for patients with agalactia [11]. Other anterior pituitary hormone deficiencies in Sheehan's syndrome may present gradually with nonspecific symptoms or may manifest suddenly and severely, such as acute adrenal insufficiency and hypothyroidism, which can be life-threatening, particularly under stressful conditions such as infection and surgery [9,10]. However, these symptoms are often mild and can be easily overlooked by the affected individual, leading to a delay in diagnosis that may extend for several years [9]. 

The mean delay in diagnosis of Sheehan’s syndrome varies significantly across reported cases [9]. In the retrospective cohort study conducted by Ramiandrasoa et al., the mean delay was found to be 9±9.7 years [11]. Another retrospective study by Gokalp et al., which included 124 women with Sheehan’s syndrome from 1995 to 2015, reported an average diagnostic delay of 20.37 ± 8.34 years [5]. In our case report, the patient experienced a diagnostic delay of 46 years, which exceeded all previous reports. This variation could be explained by the nonspecific nature of Sheehan’s syndrome and its diverse clinical presentation. Several factors have been suggested as contributing to this diagnostic delay. One theory suggests that the clinical presentation of Sheehan’s syndrome develops gradually, with patients typically exhibiting mild and vague hormonal deficiencies that are frequently mistaken for other disorders [9]. Additionally, in many cases, Sheehan's syndrome remains asymptomatic until a stressful event develops and triggers symptoms and signs of adrenal insufficiency and/or hypothyroidism [10]. Furthermore, the lack of awareness among healthcare providers and the absence of clear signs during the immediate postpartum period can contribute to a significant diagnostic delay [12].

Growth hormone and prolactin deficiencies are the most common hormonal disturbances, occurring in 90-100% of Sheehan’s syndrome cases, followed by deficiencies in gonadotrophins, thyrotrophin, and corticotrophin, which occur in 50-100% of cases [5,9]. In our case, the hormonal assay indicated deficiencies in all anterior pituitary hormones. In addition to the hormonal irregularities, Sheehan's syndrome is often associated with other laboratory abnormalities, including hyponatremia and anemia of chronic illness [8], which were also present in our patient. The hyponatremia is considered one of the most common electrolyte disturbances, reported in up to 33-69% of Sheehan’s syndrome cases [11,13]. It typically develops secondary to adrenal insufficiency or central hypothyroidism [4]. Normocytic normochromic anemia is commonly observed in patients with Sheehan's syndrome [8]. A study conducted by Gokalp et al. involving 65 patients between 1997 and 2008 revealed that 80% of these individuals had anemia, compared to only 25% of the control group [14].

In the current case, the initial diagnostic clue was the presence of symptomatic hyponatremia, which prompted further evaluation, including testing the cortisol level in the early morning and thyroid function, which revealed abnormalities in both hormonal axes. This led us to question the underlying cause of hypopituitarism in this patient. The second clue in this case was the patient's obstetric history of lactational failure following the delivery. 

Pituitary MRI can show different features based on the disease's stage [8]. In the later stages of the disease, a partial or complete empty sella is commonly observed; however, normal MRI findings cannot definitively exclude hypopituitarism [9]. For our patient, the brain MRI revealed an enlarged sella turcica and predominantly filled with cerebrospinal fluid, and the pituitary gland was significantly compressed against the sellar floor, consistent with a partially empty sella. The treatment of Sheehan's syndrome generally involves replacing the deficient hormones [9]. Hydrocortisone is typically replaced first, followed by TSH, as administering thyroid hormone before hydrocortisone may worsen glucocorticoid deficiency and potentially lead to an adrenal crisis. Growth hormone replacement in adults remains controversial [8]. We treated our patient with hydrocortisone and levothyroxine, resulting in marked improvement in her symptoms and biochemical profile.

## Conclusions

This case of an elderly woman with Sheehan's syndrome, presenting 46 years after severe postpartum hemorrhage, underscores the critical need for maintaining a high index of suspicion for endocrine disorders in patients with a history of significant obstetric complications. Despite the rarity and delayed presentation of Sheehan's syndrome, timely diagnosis and hormone replacement therapy can significantly improve patient outcomes. Early recognition and treatment are paramount to preventing life-threatening complications and improving quality of life.
